# Crystal structure of [UO_2_(NH_3_)_5_]NO_3_·NH_3_

**DOI:** 10.1107/S2056989016016480

**Published:** 2016-11-01

**Authors:** Patrick Woidy, Florian Kraus

**Affiliations:** a Anorganische Chemie, Fluorchemie, Philipps-Universität Marburg, Hans-Meerwein-Strasse 4, 35032 Marburg, Germany

**Keywords:** crystal structure, uranium(V), nitrate, ammonia, hydrogen bonding

## Abstract

Penta­ammine dioxide uranium(V) nitrate ammonia (1/1), [UO_2_(NH_3_)_5_]NO_3_·NH_3_, was obtained in the form of yellow crystals from the reaction of caesium uranyl nitrate, Cs[UO_2_(NO_3_)_3_], and uranium tetra­fluoride, UF_4_, in dry liquid ammonia. The [UO_2_]^+^ cation is coordinated by five ammine ligands. The resulting [UO_2_(NH_3_)_5_] coordination polyhedron is best described as a penta­gonal bipyramid with the O atoms forming the apices. In the crystal, numerous N—H⋯N and N—H⋯O hydrogen bonds are present between the cation, anion and solvent mol­ecules, leading to a three-dimensional network.

## Abstract 2

The title compound, [UO_2_(NH_3_)_5_]NO_3_·NH_3_, contains a penta­gonal–bipyramidal [UO_2_(NH_3_)_5_]^+^ cation, a nitrate anion and an ammonia mol­ecule of crystallization.

## Introduction – Chemical context   

Uranium chemistry in aqueous solution is dominated by the uranyl cation [UO_2_]^2+^, with the uranium atom in the hexa­valent oxidation state. The most prominent representatives are the well-known uranyl nitrates and uranyl halides. In contrast to the [UO_2_]^2+^ uranyl cation, the uranyl cation [UO_2_]^+^ with penta­valent uranium disproportionates in aqueous solution into the [U^VI^O_2_]^2+^ cation and a tetra­valent uranium species. Only under controlled conditions (Kraus *et al.*, 1949 ▸) and in organic solvents (Arnold *et al.*, 2009 ▸) are uranyl cations with penta­valent uranium observable. Here we report on the crystal structure of a U^V^ compound, [UO_2_(NH_3_)_5_]NO_3_·NH_3_, obtained from the reaction of U^IV^ with U^VI^ species in anhydrous liquid ammonia. The compound is not stable at temperatures above *ca* 238 K due to the loss of ammonia of a still unknown amount. Despite several efforts, we have not yet been able to reproduce the synthesis of the compound.

Obviously, the two uranium compounds used as educts reacted in a comproportionation reaction in order to form the U^V^ compound reported here. It is possible that the redox potentials in liquid ammonia are reversed compared to aqueous solutions, leading to a comproportionation. Such changes of electrochemical potentials are not uncommon and, for example, are known for the system Cu/Cu^+^/Cu^2+^ (Woidy *et al.*, 2015*a* ▸). However, the detailed reaction U^VI^ + U^IV^ → U^V^ is still unclear, and despite some efforts we were not able to elucidate further reaction products which must be present (*e.g.* fluoride containing ones).

## Results and discussion – Structural commentary   

**Figure 1:**
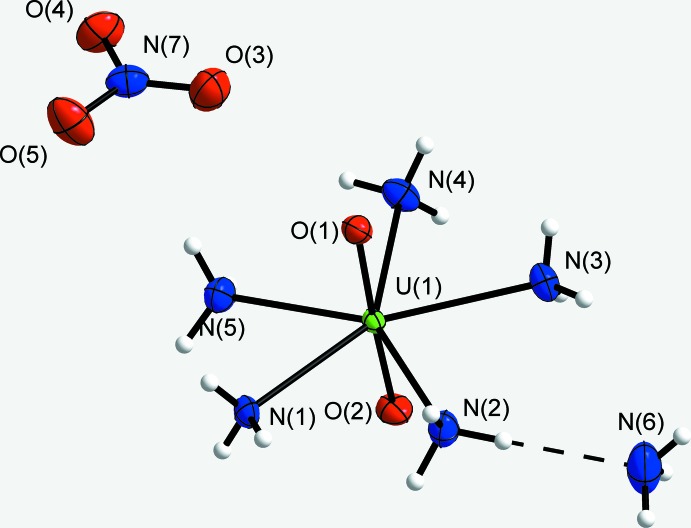
The molecular components of the title compound. Displacement ellipsoids are shown at the 70% probability level. The dashed line corresponds to a N—H⋯N hydrogen-bonding interaction.

All atoms in the structure of the title compound reside on general Wyckoff positions 8*c* of space group *Pbca*. The penta­valent uranium atom U1 and the oxygen atoms O1 and O2 form an uranyl cation. This [UO_2_]^+^ ion is coordinated by five ammine ligands (N1–N5) forming the complex penta­gonal–bipyramidal [UO_2_(NH_3_)_5_]^+^ cation which is shown in Fig. 1 ▸. The nitrate anion NO_3_^−^ consists of the nitro­gen atom N7 and the oxygen atoms O3–O5. An ammonia mol­ecule of crystallization (N6) is also observed in the structure.

The U—O distances in the almost linear uranyl cation [O—U—O angle of 177.2 (1)°] are 1.861 (3) and 1.867 (3) Å, respectively. Such distances are slightly elongated compared to reported ones for uranyl compounds with penta­valent uranium (Berthet *et al.*, 2003 ▸; Hayton & Wu, 2008 ▸), which are in the range 1.810 (4) to 1.828 (4) Å. However, U—O distances for uranyl cations [UO_2_]^2+^ with hexa­valent uranium are about 0.02 to 0.07 Å shorter. For the alkali metal uranyl nitrates, such as *M*[UO_2_(NO_3_)_3_] with *M* = K (Jouffret *et al.*, 2011 ▸; Krivovichev & Burns, 2004 ▸), Rb (Barclay *et al.*, 1965 ▸; Zalkin *et al.*, 1989 ▸) and Cs (Malcic & Ljubica, 1961 ▸), the reported U—O distances are in the range 1.746 to 1.795 Å. In uranium(VI) compounds that contain the comparable penta­ammine dioxido uranium(VI) ion [UO_2_(NH_3_)_5_]^2+^, such as [UO_2_(NH_3_)_5_]Cl_2_·NH_3_, [UO_2_F_2_(NH_3_)_3_]_2_·2NH_3_ or [UO_2_(NH_3_)_5_]Br_2_·NH_3_, U—O distances in the range 1.768 (2) to 1.771 (3) Å were reported (Woidy *et al.*, 2012 ▸, 2015*b* ▸); these are shortened by *ca* 0.1 Å compared to the uranyl ion presented here.

The nitro­gen atoms of the ammine ligands show U—N distances between 2.573 (3) and 2.629 (3) Å, which appear slightly elongated in comparison with the U—N distances determined for U^VI^ compounds such as [UO_2_(NH_3_)_5_]Cl_2_·NH_3_ [2.505 (2)–2.554 (3) Å], [UO_2_(NH_3_)_5_]Br_2_·NH_3_ or [UO_2_F_2_(NH_3_)_3_]_2_·2NH_3_ [2.522 (3) to 2.577 (3) Å] (Woidy *et al.*, 2012 ▸). In [UF_4_(NH_3_)_4_]·NH_3_ (Kraus & Baer, 2009 ▸), we observed an elongated U—N distance of 2.618 (5) Å due to the higher coordination number and different charge of the central atom.

The nitrate anion features no unexpected structural parameters and is practically identical compared to the nitrate anions of NaNO_3_ or KNO_3._ The N—O distances are 1.242 (5), 1.253 (4), and 1.254 (4) Å, the bond angles are 120° within the 3σ criterion [120.4 (3), 120.4 (3), and 119.2 (3)°] and therefore the anion is essentially planar.

As we are not able to completely explain the formation of the title compound from the educts, the question arises whether the cation is not simply a ‘regular’ uran­yl(VI) cation. It is obvious that no second nitrate anion is present in the structure. Due to chemical reasoning, the ammonia mol­ecule of crystallization also cannot be an amide anion (NH_2_^−^). As ammine ligands are bound to the uranium cation, some of their electron density is transferred to the Lewis-acidic U atom, which leads to a weakening of the N—H bonds and therefore to an acidification of these protons. So, an amide anion residing next to an acidified ammine ligand is not a plausible assumption, especially since the ammonia mol­ecule of crystallization shows an usual N⋯N distance for N—H⋯N hydrogen bonds. If one assumes that CO_3_^2−^ is present instead of NO_3_^−^, then a ‘regular’ [U^VI^O_2_]^2+^ ion would also result. However, if one refines the occupancy of the N atom of the nitrate anion, an occupancy of 1.00 (2) is observed, whereas if the occupancy of the C atom of a putative carbonate anion is refined, an occupancy of 1.30 (2) is obtained. Comparing the atomic distances of the trigonal–planar anion with the mean distances from the literature, 1.284 Å for CO_3_^2−^ (Zemann, 1981 ▸) and 1.250 Å for NO_3_^−^ (Baur, 1981 ▸), it is most likely that in our case a nitrate anion is present. In summary, all these points indicate that the central atom is an N atom of a nitrate anion. Together with the observation of slightly elongated U—O and U—N bond lengths in comparison to similar [UO_2_(NH_3_)_5_]^2+^ ions, we conclude that the compound should contain U^V^ atoms in form of [UO_2_]^+^ ions.

## Supra­molecular features   

**Figure 2:**
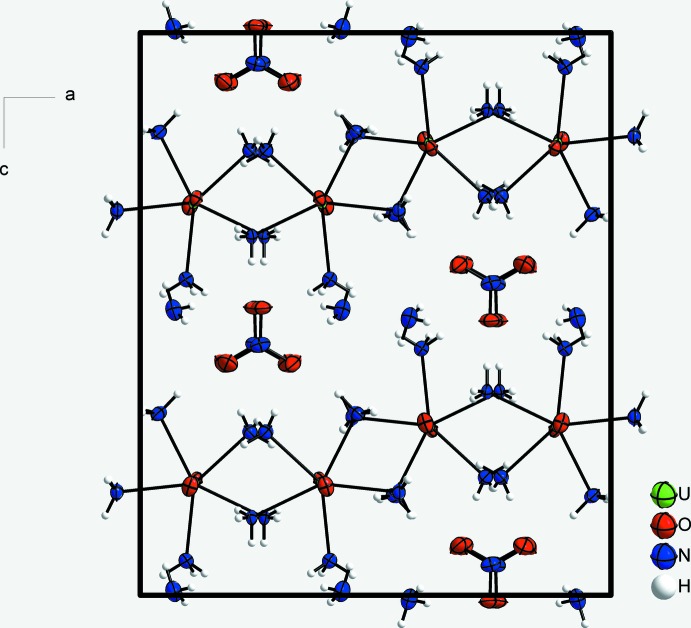
Crystal structure of [UO_2_(NH_3_)_5_]NO_3_·NH_3_ viewed along [010]. Displacement ellipsoids are shown at the 70% probability level.

The crystal structure of the title compound is shown in Fig. 2 ▸. The ammonia mol­ecule of crystallization (N6) acts as an acceptor of an N—H hydrogen bond with an ammine ligand (N2). It forms also two disparate N—H⋯O hydrogen bonds to two symmetry-equivalent nitrate anions; the third H atom (H6*C*) is not involved in hydrogen-bond formation. The nitrate anion is hydrogen-bonded to five symmetry-related [UO_2_(NH_3_)_5_]^+^ cations *via* N—H⋯O hydrogen bonds and two symmetry-related ammonia mol­ecules of crystallization. The nitrate anions lie parallel to the *ac* plane and are arranged in columns running parallel to the *b* axis (Fig. 2 ▸). The oxygen atoms of the uranyl cation act as acceptors of hydrogen bonds from four (O1) and three (O2) ammine ligands of two symmetry-related [UO_2_(NH_3_)_5_]^+^ cations. The linear UO_2_^+^ cations are also arranged parallel to the *b* axis. Overall, a three-dimensional hydrogen-bonded network results. Numerical details of the hydrogen bonding inter­actions are compiled in Table 1 ▸.

## Synthesis and crystallization   

The purity of the used educts was evidenced by powder X-ray diffraction and IR spectroscopy. 50 mg (0.09 mmol, 1 eq.) Cs[UO_2_(NO_3_)_3_] and 27 mg (0.09 mmol, 1 eq.) UF_4_ were placed in a reaction flask under argon atmosphere. After cooling to 195 K *ca* 10 ml NH_3_ were added to the reaction mixture resulting in a clear yellow solution and a green solid residue. Yellow single crystals of the title compound were obtained during storage at 233 K and were selected under cold perfluoro­ether oil (Kottke & Stalke, 1993 ▸). Additionally, emerald green crystals of [UF_4_(NH_3_)_4_]·NH_3_ were observed (Kraus & Baer, 2009 ▸) next to colourless crystals of CsNO_3_, both evidenced by determination of their unit-cell parameters.

## Refinement   

Crystal data, data collection and structure refinement details are summarized in Table 2 ▸. The structure was solved by the heavy-atom method and all other atoms were located from difference Fourier maps. In case of the hydrogen atoms of nitro­gen atoms N1–N5, their positions were refined using a riding model with N—H = 0.91 Å and *U*_eq_(H) = 1.5*U*_iso_(N). The hydrogen atoms of the ammonia mol­ecule of crystallization were refined freely. The maximum and minimum residual electron densities are located close to the U atom at distances of 0.58 and 0.04 Å, respectively.

## Supplementary Material

Crystal structure: contains datablock(s) I. DOI: 10.1107/S2056989016016480/wm5331sup1.cif

Structure factors: contains datablock(s) I. DOI: 10.1107/S2056989016016480/wm5331Isup2.hkl

CCDC reference: 1510182

Additional supporting information: crystallographic information; 3D view; checkCIF report

## Figures and Tables

**Table 1:** Hydrogen-bond geometry (Å, °)

*D*—H⋯*A*	*D*—H	H⋯*A*	*D*⋯*A*	*D*—H⋯*A*
N1—H1*A*⋯O4^i^	0.91	2.43	3.166 (4)	138
N1—H1*A*⋯O4^ii^	0.91	2.47	2.996 (4)	117
N1—H1*B*⋯O1^iii^	0.91	2.25	3.079 (4)	151
N1—H1*C*⋯O2^iv^	0.91	2.12	3.006 (4)	165
N2—H2*A*⋯O4^i^	0.91	2.49	3.220 (5)	138
N2—H2*B*⋯N6	0.91	2.14	3.024 (5)	164
N2—H2*C*⋯O4^ii^	0.91	2.36	3.232 (5)	160
N3—H3*A*⋯O2^v^	0.91	2.27	3.136 (5)	159
N3—H3*B*⋯O1^vi^	0.91	2.34	3.151 (4)	149
N3—H3*C*⋯O5^vii^	0.91	2.52	3.142 (5)	126
N4—H4*A*⋯O1^vi^	0.91	2.37	3.219 (4)	156
N4—H4*B*⋯O2^v^	0.91	2.26	3.086 (4)	150
N4—H4*C*⋯O3	0.91	2.55	3.253 (5)	134
N5—H5*A*⋯O5^iii^	0.91	2.14	3.048 (5)	176
N5—H5*B*⋯O3	0.91	2.44	3.063 (5)	126
N5—H5*B*⋯O5	0.91	2.59	3.394 (5)	147
N5—H5*C*⋯O1^iii^	0.91	2.37	3.273 (4)	171
N6—H6*A*⋯O4^vii^	0.86 (7)	2.50 (7)	3.342 (6)	167 (7)
N6—H6*B*⋯O3^vi^	0.81 (8)	2.32 (8)	3.102 (6)	162 (7)

Symmetry codes: (i) 
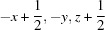
; (ii) 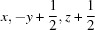; (iii) 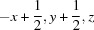; (iv) 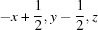; (v) 
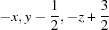
; (vi) 
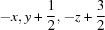
; (vii) 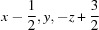.

**Table 2:** Experimental details

Crystal data	
Chemical formula	[U(NH_3_)_5_]NO_3_·NH_3_
*M*_r_	434.24
Crystal system, space group	Orthorhombic, *P* *b* *c* *a*
Temperature (K)	123
*a*, *b*, *c* (Å)	15.7497 (2), 7.7375 (1), 18.8126 (2)
*V* (Å^3^)	2292.57 (5)
*Z*	8
Radiation type	Mo *K*α
μ (mm^−1^)	14.17
Crystal size (mm)	0.24 × 0.21 × 0.04

Data collection	
Diffractometer	Oxford Diffraction Xcalibur3
Absorption correction	Multi-scan (*CrysAlis RED*; Oxford Diffraction, 2009 ▸)
*T*_min_, *T*_max_	0.166, 1.000
No. of measured, independent and observed [*I* > 2σ(*I*)] reflections	88079, 6635, 5051
*R*_int_	0.045
(sin θ/λ)_max_ (Å^−1^)	0.892

Refinement	
*R*[*F* ^2^ > 2σ(*F* ^2^)], *wR*(*F* ^2^), *S*	0.033, 0.090, 1.04
No. of reflections	6635
No. of parameters	136
H-atom treatment	H atoms treated by a mixture of independent and constrained refinement
Δρ_max_, Δρ_min_ (e Å^−3^)	5.60, −3.79

Computer programs: *CrysAlis CCD* and *CrysAlis RED* (Oxford Diffraction, 2009 ▸), *SHELXL97* (Sheldrick, 2008 ▸), *SHELXL2014* (Sheldrick, 2015 ▸), *SHELXLE* (Hübschle *et al.*, 2011 ▸), *DIAMOND* (Brandenburg, 2012 ▸) and *publCIF* (Westrip, 2010 ▸).

## supplementary crystallographic information

### Crystal data

**Table:**

[U(NH_3_)_5_]NO_3_·NH_3_	*D*_x_ = 2.516 Mg m^−^^3^
*M**_r_* = 434.24	Melting point: not measured K
Orthorhombic, *P**b**c**a*	Mo *K*α radiation, λ = 0.71073 Å
*a* = 15.7497 (2) Å	Cell parameters from 44787 reflections
*b* = 7.7375 (1) Å	θ = 2.8–39.2°
*c* = 18.8126 (2) Å	µ = 14.17 mm^−^^1^
*V* = 2292.57 (5) Å^3^	*T* = 123 K
*Z* = 8	Plate, colourless
*F*(000) = 1592	0.24 × 0.21 × 0.04 mm

### Data collection

**Table:**

Oxford Diffraction Xcalibur3 diffractometer	6635 independent reflections
Radiation source: Enhance (Mo) X-ray Source	5051 reflections with *I* > 2σ(*I*)
Graphite monochromator	*R*_int_ = 0.045
Detector resolution: 16.0238 pixels mm^-1^	θ_max_ = 39.3°, θ_min_ = 3.1°
phi– and ω–rotation scans	*h* = −27→27
Absorption correction: multi-scan (*CrysAlis RED*; Oxford Diffraction, 2009)	*k* = −8→13
*T*_min_ = 0.166, *T*_max_ = 1.000	*l* = −32→32
88079 measured reflections	

### Refinement

**Table:**

Refinement on *F*^2^	Secondary atom site location: difference Fourier map
Least-squares matrix: full	Hydrogen site location: mixed
*R*[*F*^2^ > 2σ(*F*^2^)] = 0.033	H atoms treated by a mixture of independent and constrained refinement
*wR*(*F*^2^) = 0.090	*w* = 1/[σ^2^(*F*_o_^2^) + (0.0532*P*)^2^ + 3.9914*P*] where *P* = (*F*_o_^2^ + 2*F*_c_^2^)/3
*S* = 1.04	(Δ/σ)_max_ = 0.003
6635 reflections	Δρ_max_ = 5.60 e Å^−^^3^
136 parameters	Δρ_min_ = −3.79 e Å^−^^3^
0 restraints	Extinction correction: SHELXL2014 (Sheldrick, 2015), Fc^*^=kFc[1+0.001xFc^2^λ^3^/sin(2θ)]^-1/4^
Primary atom site location: heavy-atom method	Extinction coefficient: 0.00070 (7)

### Special details

**Table:**

Geometry. All esds (except the esd in the dihedral angle between two l.s. planes) are estimated using the full covariance matrix. The cell esds are taken into account individually in the estimation of esds in distances, angles and torsion angles; correlations between esds in cell parameters are only used when they are defined by crystal symmetry. An approximate (isotropic) treatment of cell esds is used for estimating esds involving l.s. planes.

### Fractional atomic coordinates and isotropic or equivalent isotropic displacement parameters (Å^2^)

**Table:**

	*x*	*y*	*z*	*U*_iso_*/*U*_eq_	
U1	0.11450 (2)	0.22849 (2)	0.80178 (2)	0.00916 (4)	
O1	0.11836 (14)	−0.0112 (4)	0.79457 (13)	0.0145 (4)	
O2	0.10583 (16)	0.4688 (4)	0.80629 (15)	0.0180 (5)	
N1	0.26344 (17)	0.2174 (4)	0.86194 (16)	0.0140 (5)	
H1A	0.257174	0.197530	0.909327	0.021*	
H1B	0.290520	0.320039	0.855278	0.021*	
H1C	0.294646	0.130809	0.842227	0.021*	
N2	0.0977 (2)	0.2180 (5)	0.93878 (17)	0.0182 (6)	
H2A	0.111922	0.110928	0.954839	0.027*	
H2B	0.042857	0.241370	0.950589	0.027*	
H2C	0.132433	0.298114	0.959019	0.027*	
N3	−0.04957 (19)	0.2180 (4)	0.81645 (19)	0.0185 (6)	
H3A	−0.070849	0.128341	0.790693	0.028*	
H3B	−0.072525	0.318832	0.800654	0.028*	
H3C	−0.062761	0.203054	0.863146	0.028*	
N4	0.0394 (2)	0.2335 (4)	0.67697 (19)	0.0188 (6)	
H4A	0.003321	0.325238	0.674759	0.028*	
H4B	0.009622	0.133855	0.670795	0.028*	
H4C	0.079243	0.243219	0.642176	0.028*	
N5	0.2334 (2)	0.2756 (5)	0.70995 (18)	0.0190 (6)	
H5A	0.214265	0.346174	0.674728	0.029*	
H5B	0.248928	0.172025	0.691072	0.029*	
H5C	0.278944	0.325155	0.731585	0.029*	
N6	−0.0713 (3)	0.3021 (8)	1.0069 (3)	0.0327 (9)	
H6A	−0.110 (4)	0.227 (8)	1.013 (4)	0.033 (19)*	
H6B	−0.095 (4)	0.381 (10)	0.986 (4)	0.05 (2)*	
H6C	−0.056 (6)	0.312 (12)	1.037 (5)	0.06 (3)*	
N7	0.2550 (2)	0.0309 (4)	0.55493 (17)	0.0204 (6)	
O3	0.1861 (2)	0.0340 (5)	0.58716 (17)	0.0320 (7)	
O4	0.25705 (19)	0.0485 (4)	0.48871 (15)	0.0266 (6)	
O5	0.3231 (2)	0.0085 (5)	0.58813 (17)	0.0311 (7)	

### Atomic displacement parameters (Å^2^)

**Table:**

	*U*^11^	*U*^22^	*U*^33^	*U*^12^	*U*^13^	*U*^23^
U1	0.00711 (5)	0.00894 (6)	0.01145 (5)	0.00013 (3)	−0.00104 (3)	−0.00027 (3)
O1	0.0137 (10)	0.0142 (12)	0.0157 (11)	−0.0007 (8)	−0.0016 (8)	−0.0011 (8)
O2	0.0176 (11)	0.0096 (11)	0.0267 (14)	0.0021 (8)	−0.0032 (9)	−0.0019 (9)
N1	0.0093 (10)	0.0178 (14)	0.0147 (11)	0.0020 (9)	−0.0012 (8)	−0.0006 (10)
N2	0.0140 (11)	0.0250 (16)	0.0156 (12)	0.0012 (10)	0.0010 (10)	−0.0005 (11)
N3	0.0111 (11)	0.0222 (16)	0.0221 (14)	−0.0006 (10)	−0.0004 (10)	0.0003 (11)
N4	0.0184 (13)	0.0214 (15)	0.0167 (12)	−0.0025 (11)	−0.0054 (11)	0.0006 (11)
N5	0.0167 (13)	0.0230 (16)	0.0172 (13)	−0.0017 (11)	0.0026 (10)	−0.0014 (11)
N6	0.0188 (15)	0.049 (3)	0.030 (2)	0.0031 (17)	0.0034 (15)	0.002 (2)
N7	0.0263 (16)	0.0193 (15)	0.0158 (13)	−0.0001 (12)	−0.0030 (11)	−0.0010 (11)
O3	0.0275 (14)	0.044 (2)	0.0242 (15)	0.0026 (14)	0.0024 (12)	−0.0076 (14)
O4	0.0347 (16)	0.0330 (17)	0.0121 (11)	0.0024 (13)	−0.0015 (11)	0.0020 (11)
O5	0.0269 (14)	0.0409 (19)	0.0255 (15)	−0.0051 (13)	−0.0095 (12)	0.0083 (14)

### Geometric parameters (Å, º)

**Table:**

U1—O1	1.861 (3)	N3—H3B	0.9100
U1—O2	1.867 (3)	N3—H3C	0.9100
U1—N5	2.573 (3)	N4—H4A	0.9100
U1—N2	2.592 (3)	N4—H4B	0.9100
U1—N3	2.600 (3)	N4—H4C	0.9100
U1—N1	2.606 (3)	N5—H5A	0.9100
U1—N4	2.629 (3)	N5—H5B	0.9100
N1—H1A	0.9100	N5—H5C	0.9100
N1—H1B	0.9100	N6—H6A	0.86 (7)
N1—H1C	0.9100	N6—H6B	0.81 (8)
N2—H2A	0.9100	N6—H6C	0.63 (9)
N2—H2B	0.9100	N7—O3	1.242 (5)
N2—H2C	0.9100	N7—O5	1.253 (4)
N3—H3A	0.9100	N7—O4	1.254 (4)
			
O1—U1—O2	177.20 (11)	H2A—N2—H2B	109.5
O1—U1—N5	93.92 (11)	U1—N2—H2C	109.5
O2—U1—N5	86.72 (12)	H2A—N2—H2C	109.5
O1—U1—N2	92.56 (11)	H2B—N2—H2C	109.5
O2—U1—N2	88.76 (12)	U1—N3—H3A	109.5
N5—U1—N2	138.26 (11)	U1—N3—H3B	109.5
O1—U1—N3	90.52 (11)	H3A—N3—H3B	109.5
O2—U1—N3	87.34 (11)	U1—N3—H3C	109.5
N5—U1—N3	143.01 (11)	H3A—N3—H3C	109.5
N2—U1—N3	78.00 (11)	H3B—N3—H3C	109.5
O1—U1—N1	88.25 (10)	U1—N4—H4A	109.5
O2—U1—N1	94.52 (10)	U1—N4—H4B	109.5
N5—U1—N1	68.98 (10)	H4A—N4—H4B	109.5
N2—U1—N1	70.06 (10)	U1—N4—H4C	109.5
N3—U1—N1	147.94 (10)	H4A—N4—H4C	109.5
O1—U1—N4	87.96 (11)	H4B—N4—H4C	109.5
O2—U1—N4	89.59 (11)	U1—N5—H5A	109.5
N5—U1—N4	74.08 (11)	U1—N5—H5B	109.5
N2—U1—N4	147.40 (11)	H5A—N5—H5B	109.5
N3—U1—N4	69.40 (11)	U1—N5—H5C	109.5
N1—U1—N4	142.49 (10)	H5A—N5—H5C	109.5
U1—N1—H1A	109.5	H5B—N5—H5C	109.5
U1—N1—H1B	109.5	H6A—N6—H6B	104 (7)
H1A—N1—H1B	109.5	H6A—N6—H6C	103 (10)
U1—N1—H1C	109.5	H6B—N6—H6C	121 (10)
H1A—N1—H1C	109.5	O3—N7—O5	120.4 (3)
H1B—N1—H1C	109.5	O3—N7—O4	120.4 (3)
U1—N2—H2A	109.5	O5—N7—O4	119.2 (3)
U1—N2—H2B	109.5		

### Hydrogen-bond geometry (Å, º)

**Table:**

*D*—H···*A*	*D*—H	H···*A*	*D*···*A*	*D*—H···*A*
N1—H1*A*···O4^i^	0.91	2.43	3.166 (4)	138
N1—H1*A*···O4^ii^	0.91	2.47	2.996 (4)	117
N1—H1*B*···O1^iii^	0.91	2.25	3.079 (4)	151
N1—H1*C*···O2^iv^	0.91	2.12	3.006 (4)	165
N2—H2*A*···O4^i^	0.91	2.49	3.220 (5)	138
N2—H2*B*···N6	0.91	2.14	3.024 (5)	164
N2—H2*C*···O4^ii^	0.91	2.36	3.232 (5)	160
N3—H3*A*···O2^v^	0.91	2.27	3.136 (5)	159
N3—H3*B*···O1^vi^	0.91	2.34	3.151 (4)	149
N3—H3*C*···O5^vii^	0.91	2.52	3.142 (5)	126
N4—H4*A*···O1^vi^	0.91	2.37	3.219 (4)	156
N4—H4*B*···O2^v^	0.91	2.26	3.086 (4)	150
N4—H4*C*···O3	0.91	2.55	3.253 (5)	134
N5—H5*A*···O5^iii^	0.91	2.14	3.048 (5)	176
N5—H5*B*···O3	0.91	2.44	3.063 (5)	126
N5—H5*B*···O5	0.91	2.59	3.394 (5)	147
N5—H5*C*···O1^iii^	0.91	2.37	3.273 (4)	171
N6—H6*A*···O4^vii^	0.86 (7)	2.50 (7)	3.342 (6)	167 (7)
N6—H6*B*···O3^vi^	0.81 (8)	2.32 (8)	3.102 (6)	162 (7)

Symmetry codes: (i) −*x*+1/2, −*y*, *z*+1/2; (ii) *x*, −*y*+1/2, *z*+1/2; (iii) −*x*+1/2, *y*+1/2, *z*; (iv) −*x*+1/2, *y*−1/2, *z*; (v) −*x*, *y*−1/2, −*z*+3/2; (vi) −*x*, *y*+1/2, −*z*+3/2; (vii) *x*−1/2, *y*, −*z*+3/2.

## References

[bb1] Arnold, P. L., Love, J. B. & Patel, D. (2009). *Coord. Chem. Rev.* **253**, 1973–1978.

[bb2] Barclay, G. A., Sabine, T. M. & Taylor, J. C. (1965). *Acta Cryst.* **19**, 205–209.

[bb3] Baur, W. H. (1981). *Interatomic Distance Predictions for Computer Simulation of Crystal Structures*, in *Structure and Bonding in Crystals*, Vol. II, p. 31 ff, edited by M. O’Keeffe & M. Navrotsky. New York: Academic Press.

[bb4] Berthet, J.-C., Nierlich, M. & Ephritikhine, M. (2003). *Angew. Chem. Int. Ed.* **42**, 1952–1954. 10.1002/anie.200250506 12730979

[bb5] Brandenburg, K. (2012). *DIAMOND*. Crystal Impact GbR, Bonn, Germany.

[bb6] Hayton, T. W. & Wu, G. (2008). *J. Am. Chem. Soc.* **130**, 2005–2014. 10.1021/ja077538q 18205356

[bb7] Hübschle, C. B., Sheldrick, G. M. & Dittrich, B. (2011). *J. Appl. Cryst.* **44**, 1281–1284. 10.1107/S0021889811043202 PMC3246833 22477785

[bb8] Jouffret, L. J., Krivovichev, S. V. & Burns, P. C. (2011). *Z. Anorg. Allg. Chem.* **637**, 1475–1480.

[bb9] Kottke, T. & Stalke, D. (1993). *J. Appl. Cryst.* **26**, 615–619.

[bb10] Kraus, F. & Baer, S. A. (2009). *Chem. Eur. J.* **15**, 8269–8274. 10.1002/chem.200901044 19585645

[bb11] Kraus, K. A., Nelson, F. & Johnson, G. L. (1949). *J. Am. Chem. Soc.* **71**, 2510–2517.

[bb12] Krivovichev, S. V. & Burns, P. C. (2004). *Radiochemistry*, **46**, 16–19.

[bb13] Malcic, S. S. & Ljubica, L. M. (1961). *Bull. Boris Kidric. Inst. Nucl. Sci.* **11**, 135–139.

[bb14] Oxford Diffraction (2009). *CrysAlis CCD* and *CrysAlis RED*. Oxford Diffraction Ltd, Abingdon, England.

[bb22] Sheldrick, G. M. (2008). *Acta Cryst* A**64**, 112–122. 10.1107/S0108767307043930 18156677

[bb15] Sheldrick, G. M. (2015). *Acta Cryst.* C**71**, 3–8.

[bb16] Westrip, S. P. (2010). *J. Appl. Cryst.* **43**, 920–925.

[bb17] Woidy, P., Bühl, M. & Kraus, F. (2015*b*). *Dalton Trans.* **44**, 7332–7337. 10.1039/c5dt00180c 25797497

[bb18] Woidy, P., Karttunen, A. J. & Kraus, F. (2012). *Z. Anorg. Allg. Chem.* **638**, 2044–2052.

[bb19] Woidy, P., Karttunen, A. J., Widenmeyer, M., Niewa, R. & Kraus, F. (2015*a*). *Chem. Eur. J.* **21**, 3290–3303. 10.1002/chem.201406136 25581022

[bb20] Zalkin, A., Templeton, L. K. & Templeton, D. H. (1989). *Acta Cryst.* C**45**, 810–811.

[bb21] Zemann, J. (1981). *Fortschr. Mineral.* **59**, 95–116.

